# Fibrodysplasia ossificans progressiva: A rare disease with spinal deformity and severe hip dysfunction

**DOI:** 10.3389/fped.2022.981372

**Published:** 2022-09-15

**Authors:** Dong Sun, Peng Liu, Zhaolin Wang, Jianhu Mu, Jian Cao

**Affiliations:** Department of Orthopedics, China-Japan Union Hospital of Jilin University, Changchun, China

**Keywords:** fibrodysplasia ossificans progressive, iliopsoas muscle, heterotopic ossification, fascial release, hip dysfunction

## Abstract

**Introduction:**

Progressive fibrous dysplasia ossification (FOP) is a rare genetic disease characterized by congenital bone malformations and soft tissue masses that progress to heterotopic ossification. Congenital great toe deformity and progressive heterotopic ossifications with an anatomical and temporal pattern are the two classical clinical characteristics of FOP. We present a unique case of FOP characterized by mandibular angle fascial contracture and back and iliopsoas muscle ossification managed *via* surgery in a 13 year old girl.

**Case presentation:**

A 13 year old girl with a history of right cervical fascial release surgery and back heterotopic osteotomy presented to our clinic due to recurrence of heterotopic ossification, scoliosis, and progressive joint stiffness. Computed tomography (CT) or magnetic resonance imaging (MRI) examination confirmed heterotopic ossification of the left back and left iliopsoas muscle and spinal scoliosis. Two years after the surgery, the patient presented with recurrence of back heterotopic ossification and rapidly advancing ossification of the left iliopsoas muscle. Six months after surgery, the patient had no disability, pain and clinical recurrence, and the joint function recovered.

**Conclusions:**

In patients with multiple-site heterotopic ossification caused by FOP, oral function and hip stiffness improve with detailed facial release surgery and rehabilitation treatment. However, dorsal fascia ossification and spinal scoliosis can recur shortly after resection.

## Introduction

Fibrodysplasia ossificans progressiva (FOP) is a rare connective tissue genetic disease characterized by congenital big toe deformity and irreversible heterotopic ossification of soft tissue (1, 2). The prevalence of FOP is about 1/2,000,000, and the condition is not affected by ethnicity, geographic predisposition, and sex (3). Heterotopic bone formation leads to joint locking, making movement impossible. FOP progresses with ossification episodes. This extraskeletal bone formation is exacerbated by small soft tissue traumas, myotonia, fatigue, intramuscular injections, and influenza-like infections (4). Thus far, there is no effective preventive or treatment method for FOP. Although surgical treatment is successful in rare cases, new bone formation is observed postoperatively at the surgical site (2). Herein, we present a 13-year-old girl with FOP who presented with heterotopic ossification causing mandibular angle deformity, scoliosis, and severe hip movement limitation.

## Case presentation

**FIGURE 1:**
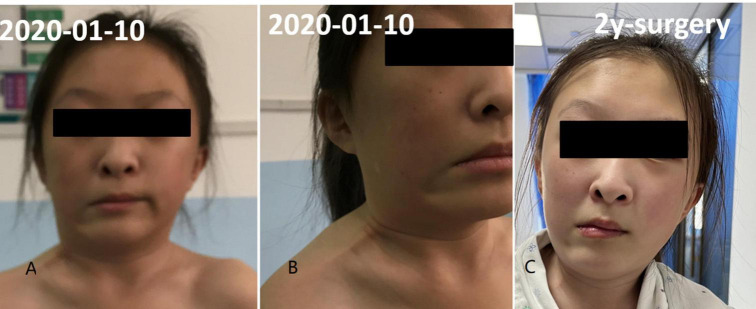
Fascial contracture of right neck and mandibular angle. Panels **(A,B)** was preoperative condition and panel **(C)** was 2 years after operation.

**FIGURE 2:**
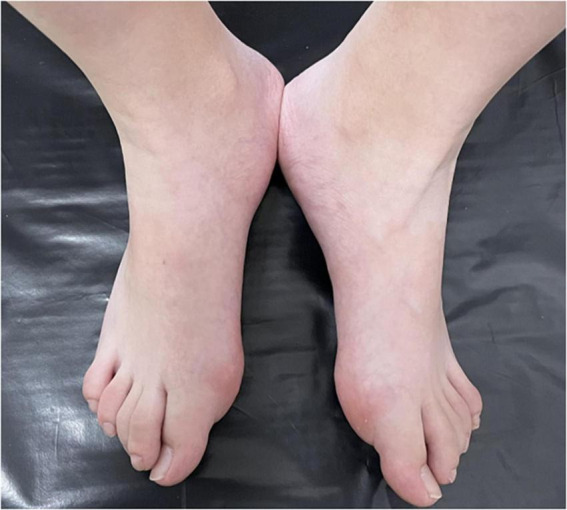
Halluces were characterized by hallux valgus deformity with macrodactyly.

**FIGURE 3:**
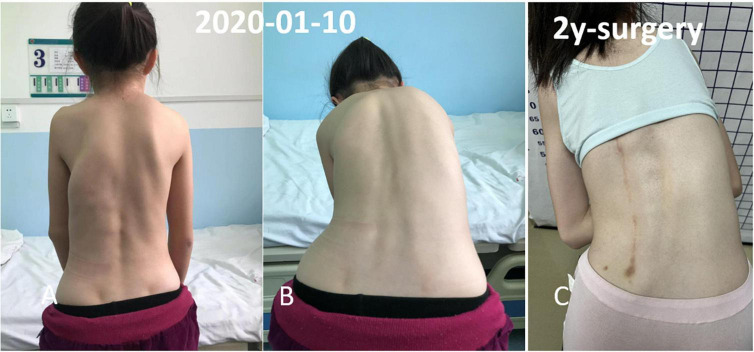
Multiple irregular, non-tender, bony hard swellings were found on the back and spinal scoliosis. The osseous lesion beginning from the left chest wall, extending to the ilium region. **(A,B)** Preoperative physical examination. **(C)** Two year follow-up.

**FIGURE 4:**
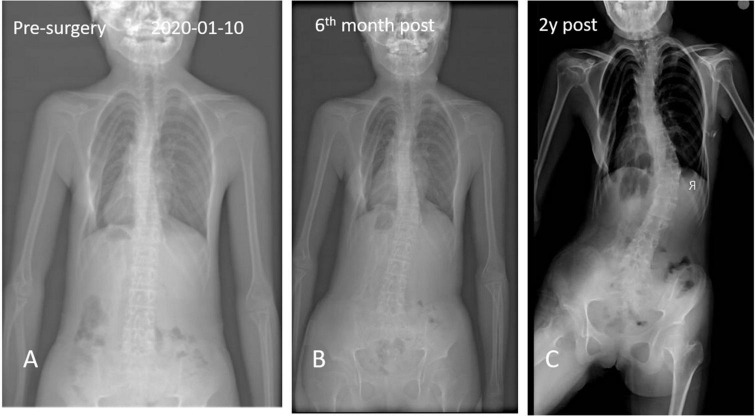
Anteroposterior and axial x-ray radiographic evaluation showed a massive radiopaque formation located predominantly in the region of back and spinal scoliosis.

**FIGURE 5:**
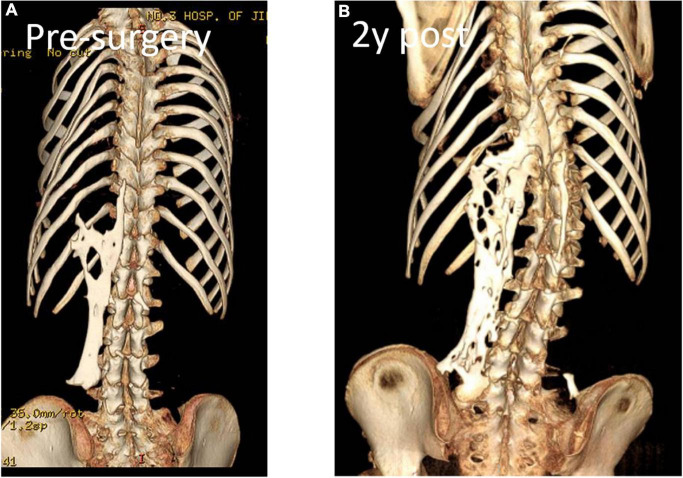
Computed tomography revealed spontaneous cervical fusion, heterotopic ossification of the left dorsal fascia causing scoliosis, and developmental deformity of the right chest wall.

**FIGURE 6:**
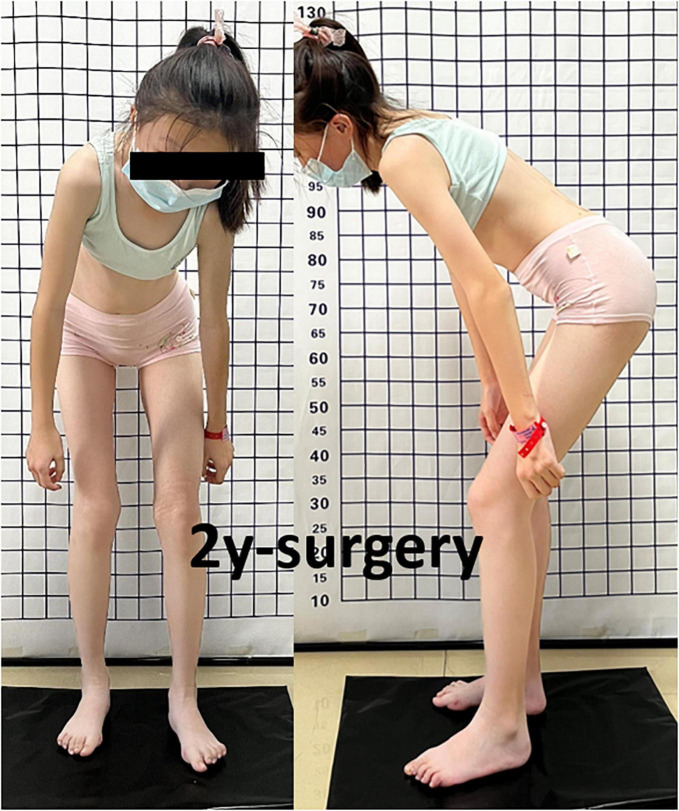
Passive left hip ROM was restricted to 80° of flexion, 5° of external rotation, and 15° of abduction with no abnormal gait pattern.

**FIGURE 7:**
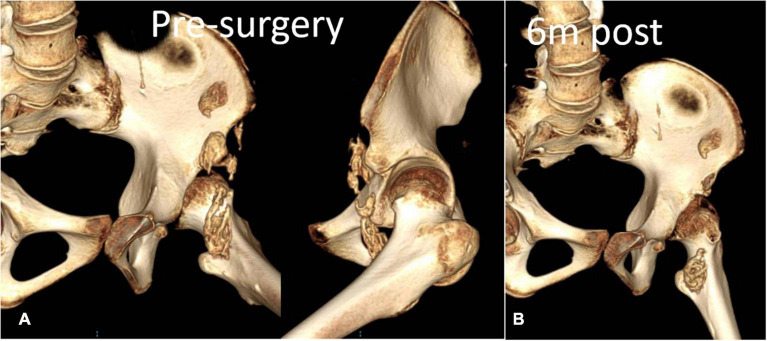
Anteroposterior view of the Computed tomography scan of the left hip confirmed aforementioned findings and additionally revealed heterotopic ossification of the iliopsoas muscle. **(A)** Preoperative CT reconstruction. **(B)** 6-month follow-up.

**FIGURE 8:**
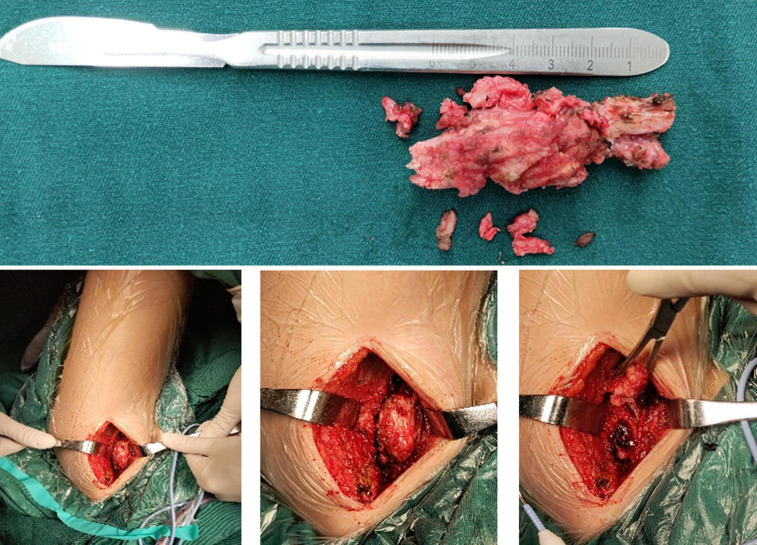
Intraoperative removed ossification mass.

**FIGURE 9:**
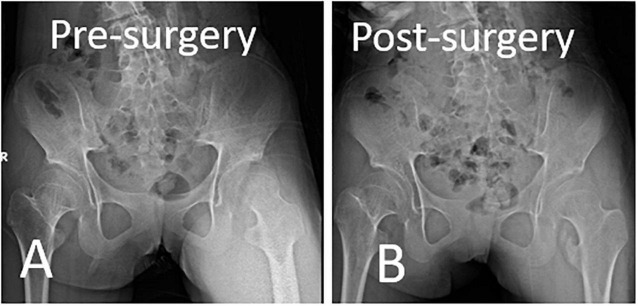
Immediate postoperative anteroposterior pelvis x-ray showed tailored removal of ossification mass according to the preoperative plan. **(A)** Preoperative X-ray. **(B)** Immediate postoperative X-ray.

A 13-year-old girl who had been experiencing spinal scoliosis and rapidly advancing hip stiffness for 2 years visited our hospital. Two years back, she was admitted due to drooping of the right corner of the mouth and ossification of the back fascia (Figures 1A,B, 2, 3A,B). Physical examination revealed facial asymmetry, palpable tumors in the left mandibular angle, and limited oral closure. However, pain was not observed. At the age of 8 years, the patient developed soft tissue heterotopic ossification in the back. After several days, the lesion became stiff like a stone. Halluces were characterized by hallux valgus deformity with big toe deformity. The patient did not have a previous history of trauma, surgeries, infections, allergies, other known underlying conditions, and medication use. She is the second child of a healthy non-consanguineous marriage and has a healthy 24-year-old sister. Her mother remembered that her grandmother had microdactyly of the hallux but no extraskeletal bone formation on the body and no signs of limited activity. Anteroposterior and axial radiographic evaluation showed that a large amount of radiopaque formation was mainly located in the back area and scoliosis (Figures 4A,B). There was a stiff, osseous lesion originating from the left chest wall and extending to the ilium region (Figure 3B). Computed tomography scan revealed spontaneous cervical fusion, heterotopic ossification of the left dorsal fascia causing scoliosis, and developmental deformity of the right chest wall (Figure 5A). The Risser’s sign was grade 0. The patient was then diagnosed with FOP according to the presence of congenital great toe deformity, dorsal fascia heterotopic ossification, and cervical fascia contracture. Therefore, genetic testing was recommended to obtain a definite diagnosis. However, due to financial constraints, the examination was not performed. To prevent the progression of scoliosis caused by tethering during the peak period of spinal growth and oral closure difficulties, back fascia ossification resection and fascial release surgery, which is a minimally invasive procedure, were performed. After 6 months of follow-up, the right cervical fascial contracture, closing movement of the mouth, and facial asymmetry significantly improved. However, the patient developed soft tissue ossification in the back. After several days, the swollen area progressed to ossification. After 2 years of follow-up, the patient came to our hospital for treatment due to continuous right hip pain and rapid progress of hip stiffness, which developed within the last 2 months. Passive left hip range of motions (ROMs) were restricted to 80° flexion, 5° external rotation, and 15° abduction with abnormal gait pattern (Figure 6). She had a history of snoring for 3 years. Physical examination showed improved facial asymmetry and mandibular angle fascia contracture and a mass in the left iliopsoas muscle with bulging but intact overlying skin (Figures 1C, 3C). The mass originated from the left medial side of the iliac and extended to the lesser trochanter on the left leg region (Figures 7A, 9A). Anteroposterior radiography and computed tomography scan showed recurrent heterotopic ossification of the left back with spinal scoliosis and chest wall malformations (Figures 4C, 5B). The patient was provided with a detailed explanation of her medical condition and treatment options. Based on history taking, clinical examination, and thorough diagnostic investigations, surgical resection of large heterotopic ossification in the left iliopsoas muscle area. However, the heterotopic ossification in the left back was left because the growth in the spine stopped. We performed careful dissection to reach the edge of the heterotopic ossified mass, thereby preventing any damage to the neurovascular supply (Figure 8). Resection of the iliopsoas ossification was conducted, and the ossification distal to the lesser trochanter was left to prevent vascular and nerve injury (Figure 8). Intraoperative blood loss 50 ml. Apply drainage tube and confirm again that there is no limitation of joint movement before suturing the wound. Immediately after operation, anteroposterior pelvis radiography showed successful resection of heterotopic ossified mass, which was in accordance with the preoperative plan (Figure 9B). We administrated indomethacin for 4 weeks and thromboprophylaxis for 3 weeks (5, 6). The patient was discharged 7 days after operation, the pain was tolerable, and the passive left ROM was unobstructed. She walked without crutches and was advised to avoid any intense physical activity (e.g., strenuous hip and stretching exercises) for another 2 months. During the follow-up of 6 months after operation, no signs of disability, hip pain and infection were observed, and there were no radiological indications of ossification recurrence (Figure 7B). The passive left hip ROMs were 100° flexion, 35° external rotation, 10° internal rotation, and 30° abduction.

## Discussion

Here, we report a special case characterized by fascial contracture of the mandibular horn and back and iliopsoas muscle ossification managed *via* surgery in a 13-year-old girl. The surgical effect was confirmed during follow-up. Two years after the surgery, mandibular angle fascia contracture and joint stiffness caused by iliopsoas muscle ossification significantly improved. However, back fascia ossification recurred after 6 months.

FOP is a rare genetic disease, which is characterized by congenital bone malformation and soft tissue mass progression to heterotopic ossification. In 1740, John freke first described progressive ossifying fibrous dysplasia (MIM 135100). In order to understand the primary connective tissue involvement of tendons, ligaments, fascia and aponeurosis, Bauer and Bode proposed the term “progressive ossifying fibrous dysplasia” in 1940 and was adopted by Mccussy in 1960 (7). Most patients with FOP have the same ACVR1 gene mutation (c.617g > a; r206h) and classic clinical features. Other mutation sites include L196P, R258S, P197/F198lL, R202I, Q207E, G325A, G328W/G328E/G328R, G356D and R375 (7–15).

Heterotrophic ossification commonly begins in the first decade of the patient’s life. FOP is not associated with ethnicity, race, sex, and geographic predisposition (3, 16, 17). FOP patients have normal characteristics at birth, except for big toe deformities such as pseudoepiphysis, abnormal segmentation, first metatarsal fusion, and hallux valgus (18, 19). In the first 10 years of life, they may develop sporadic painful soft tissue swelling (flare-up) and gradual transformation of the skeletal muscle, tendon, fascia, or aponeurosis into heterotopic ossification These symptoms may occur spontaneously or may be caused by minor trauma, such as muscle stretching, muscular injection, falls, and infections. In a study of 500 patients with FOP, the neck, upper back and shoulder were the first affected areas, with a median age of 8.5–11.5 years. Heterotopic ossification starts from the axis and extends distally to the appendicular area and from the upper limb to the lower limb area, and the latest affected areas are fingers and feet. Chest wall involvement and spinal deformities, including kyphosis, thoracic lordosis and scoliosis, cause thoracic insufficiency syndrome, leading to repeated respiratory tract infections and cardiothoracic failure (20, 21).

In the current case, imaging examination of bilateral short and wide femoral neck and cervical fusion. Moreover, plain radiography revealed high-density tissue ossification on the back and bony bridges of the iliopsoas muscle. High-density muscle calcification and partial fusion with the adjacent bone were observed on computed tomography scans. Kaplan et al. proposed a diagnostic criteria for FOP (22). That is, patients should present with not only clinical and imaging but also definitive genetic characteristics (ACVR1 gene mutation). Therefore, genetic testing was recommended to obtain a definite diagnosis. However, due to financial constraints, the examination was not performed.

Thus far, there is no effective treatment method for FOP. It is necessary to reduce trauma, modify daily activities to an acceptable level, use instruments that can reduce the incidence of falls and injuries, and prevent sports that may cause tissue damage and muscle fatigue. Despite the lack of clinical evidence, brief oral corticosteroid treatment within the first 24 h can inhibit seizures (23). Non-steroidal anti-inflammatory drugs, narcotic analgesics, mast cell and leukotriene inhibitors, and bisphosphate drugs can also be used during or after the attack. However, there is no evidence to support the effect of these drugs on FOP lesions. In the past decade, with the continuous improvement of the understanding of the pathogenesis of FOP, new potential drug targets, such as abnormal regulation of BMP signal, new functions of mutant receptors, the differentiation process of cartilage formation, and the destruction of acvr1/alk-2 expression in transcription and hypoxia regulation around the microenvironment of focal lesions, have been found to be useful in the treatment of FOP (23).

To our knowledge, this is the first case of non-traumatic massive heterotopic iliopsoas ossification described in the literature. Hip joint function and oral closure improved after surgery and rehabilitation treatment. In patients with multiple-site heterotopic ossification caused by FOP, oral function and hip stiffness improve with detailed facial release surgery and rehabilitation treatment. However, dorsal fascia ossification and spinal scoliosis can recur shortly after resection.

## Data availability statement

The original contributions presented in this study are included in the article/supplementary material, further inquiries can be directed to the corresponding author.

## Ethics statement

The studies involving human participants were reviewed and approved by the Human Ethics Committee of China–Japan Union Hospital of Jilin University. Written informed consent was obtained from the individual(s), and minor(s)’ legal guardian/next of kin, for the publication of any potentially identifiable images or data included in this article.

## Author contributions

DS designed the study, conducted all searches, appraised all potential studies, and wrote and revised the draft manuscript and subsequent manuscripts. PL revised the draft manuscript and subsequent manuscripts. JM and ZW assisted with the presentation of findings and assisted with drafting and revising the manuscript. JC conceived and designed the study, assisted with searches, appraised relevant studies and assisted with drafting and revising the manuscript. All authors read and approved the final manuscript.

## Abbreviations

FOP: Fibrodysplasia Ossificans Progressiva
ROM: range of motion.
